# Quality of life during and following sequential treatment of previously untreated patients with multiple myeloma: findings of the Medical Research Council Myeloma IX randomised study

**DOI:** 10.1111/bjh.15459

**Published:** 2018-07-09

**Authors:** Kara‐Louise Royle, Walter M. Gregory, David A. Cairns, Sue E. Bell, Gordon Cook, Roger G. Owen, Mark T. Drayson, Faith E. Davies, Graham H. Jackson, Gareth J. Morgan, J. Anthony Child

**Affiliations:** ^1^ CTRU University of Leeds Leeds UK; ^2^ LICAP University of Leeds Leeds UK; ^3^ HMDS St James's University Hospital Leeds UK; ^4^ Clinical Immunology Service University of Birmingham Birmingham UK; ^5^ Institute of Cancer Research Sutton UK; ^6^ Northern Cancer Centre Newcastle University Newcastle‐upon‐Tyne UK; ^7^ Present address: Myeloma Institute University of Arkansas for Medical Sciences Little Rock AR USA

**Keywords:** multiple myeloma, quality of life, EORTC QLQ‐C30, EORTC MY‐24, immunomodulatory agent

## Abstract

In the Medical Research Council (MRC) Myeloma IX trial (ISRCTN684564111) patients were randomised to sodium clodronate or zoledronic acid and induction treatment: cyclophosphamide, vincristine, doxorubicin and dexamethasone (CVAD) or cyclophosphamide, thalidomide and dexamethasone (CTD) followed by autologous stem cell transplant (ASCT) in the intensive pathway; attenuated CTD or melphalan and prednisolone (MP) in the non‐intensive pathway. Subsequent randomisation allocated patients to either thalidomide or observation. The European Organisation for Research and Treatment of Cancer (EORTC) quality of life (QoL) questionnaires, QLQ‐C30 and QLQ‐MY24, were administered at baseline, 3, 6 and 12 months and annually thereafter, enabling the effect of sequential treatment on patient‐reported health‐related QoL (HR‐QoL) to be investigated. The protocol specified four subscales of interest: Pain, Fatigue, Global Health Status/Quality of Life and Physical Functioning at 3, 6 and 12 months that were compared using linear models. The intensive pathway showed significant differences in favour of CTD for Fatigue at 3 months and Physical Functioning at 12 months. The non‐intensive pathway and maintenance phase reported significant differences at 3 months; Pain (improved with attenuated CTD) and Global Health status/Quality of Life (improved with observation). The improved outcomes in MRC Myeloma IX were accompanied by some beneficial and few detrimental effects on HR‐QoL.

The widespread adoption of intensive therapy and the subsequent introduction of novel agents in the treatment of multiple myeloma (MM) inevitably raises questions as to effects on health‐related quality of life (HR‐QoL). However, there is still relatively little published quality of life (QoL) data in systematically treated and assessed patients, notably in the first‐line treatment setting.

The Medical Research Council (MRC) series of randomised controlled trials of treatment in previously untreated patients with MM up to MRC Myeloma IX provided only limited data on performance status. Myeloma IX included intensive and non‐intensive pathways and enrolled patients of all ages. Patient‐reported HR‐QoL was a secondary endpoint in the trial protocol, with the aim of obtaining QoL data during the induction, consolidation and maintenance phases of treatment to assess the impact of the different regimens and components of treatment. Here, we present the results of the longitudinal analyses.

## Methods

### MRC myeloma IX

Myeloma IX recruited patients with newly‐diagnosed MM aged ≥18 years from 120 centres in the United Kingdom between 2003 and 2007 and was registered with the appropriate organisation (ISRCTN68454111). All 120 centres reviewed the protocol and all recruited participants gave written informed consent in accordance with the Declaration of Helsinki, with an opt‐in component for the QoL sub‐study. The study was an open‐label randomised, phase III trial with a factorial design in which there were two randomisations. The first included randomised allocation of patients to receive a bisphosphonate (sodium clodronate or zoledronic acid) and induction treatment, either intensive or non‐intensive, determined by the treating clinicians’ assessments in combination with patient performance status and preferences following informed discussion. In the intensive pathway, patients were randomised to receive cyclophosphamide, vincristine, doxorubicin and dexamethasone (CVAD) or oral cyclophosphamide, thalidomide and dexamethasone (CTD) followed by high dose melphalan plus autologous stem cell transplant (ASCT). In the non‐intensive pathway, the randomisation was to attenuated CTD (CTDa) or melphalan and prednisolone (MP). A further randomisation allocated patients to either thalidomide maintenance therapy or observation only. Details of the trial, including the randomisation methods and sample size calculation, have been published elsewhere together with results of primary and secondary endpoints related to treatment effectiveness and safety (Morgan *et al*, 2010, 2012a,b,c, 2013).

### Patient‐reported outcome data collection

To collect patient‐reported QoL information, two patient‐completed European Organisation for Research and Treatment of Cancer (EORTC) questionnaires were used: the EORTC QLQ‐C30 and QLQ‐MY24. The QLQ‐C30 is a fully validated tool used for assessing the QoL of cancer patients (Aaronson *et al*, 1993). When scored, the 30 questions produce 15 subscales relating to HR‐QoL. The QLQ‐MY24 is a specific questionnaire module for patients with MM (Stead *et al*, 1999). The QLQ‐MY24 was validated throughout the original study period and consists of 24 questions, which when scored results in 5 subscales. As a result of the validation process the QLQ‐MY24 has been refined to the QLQ‐MY20 (removing the Social Support subscale) (Cocks *et al*, 2007). However, as it was administered, the original QLQ‐MY24 is used here.

The over‐arching QoL population was defined as any patient in the intention to treat (ITT) population, who returned a questionnaire at any time point. The protocol stated that consenting patients should complete questionnaires at baseline, 3, 6, 12 months and then annually up to 5 years post‐randomisation, unless the patient participated in the maintenance randomisation when they should complete a pre‐maintenance randomisation questionnaire and then at the same intervals as above. For the primary analysis three separate QoL populations were considered; intensive, non‐intensive and maintenance. The intensive population was defined as any individual who returned a questionnaire during their induction treatment (pre‐second randomisation) and was randomised to receive CTD or CVAD. Similarly, the non‐intensive population was defined as any individual who returned a questionnaire during their induction treatment (pre‐second randomisation) and was randomised to receive CTDa or MP. Finally, the maintenance population was defined as any individual who was randomised to receive maintenance therapy (thalidomide or observation only) and returned a questionnaire during their treatment. In all three populations, questionnaire compliance was calculated as the proportion of those in the population who returned a questionnaire out of those expected to return a questionnaire (alive on study treatment and not withdrawn). An alternative population to the non‐intensive and intensive populations was defined for some of the exploratory analysis. This considered the two populations together as a first randomisation population; specifically, any individual who returned a questionnaire during their induction treatment (pre‐second randomisation) was included in this population.

### Data imputation and subscale scoring

Missing questionnaire data and relevant baseline factors were imputed using multiple imputation by chained equations (MICE) (White *et al*, 2011). The imputation was conducted separately for the three analysis populations described above. In addition, the imputation was conducted by randomised induction treatment in the intensive and non‐intensive pathways and randomised treatment in the maintenance phase, therefore only individuals with the same randomisation allocation contributed to an individual's imputed value.

The total number of imputed datasets was determined as the maximum percentage of ‘missingness’ in a compulsory question at any time point, or baseline measurement [stratification factors, age, sex and International Staging System (ISS)] across the three populations. For each population, imputation was conducted in temporal order, therefore the baseline measurements were imputed first, followed by baseline questionnaire values, then the 3 months questionnaire values and so on. Within each time point, the variables were imputed in order of least to most missing. Note that only questionnaires from the treatment period being considered were used within each imputation phase: for example, only induction questionnaires from non‐intensive pathway patients were used to impute data for the non‐intensive pathway.

The imputed datasets were scored according to the questionnaire manuals and analysis performed in each imputed dataset. The parameter estimates (coefficient and standard errors) were combined according to Rubin (1987) and appropriate Wald tests obtained (White *et al*, 2011). All analyses was undertaken using SAS version 9.4 (SAS Institute, Cary, NC, USA) and data collected up to 8 May 2014.

### Primary analysis

Analyses of patient‐reported QoL were pre‐specified in a separate statistical analysis plan. For the primary analysis the dependent variables were defined as the four subscales of interest (Pain, Fatigue, Global Health Status/Quality of Life and Physical Functioning) at three time points (3, 6 and 12 months) for each analysis population (intensive, non‐intensive and maintenance).

Multiple linear regression was used to regress the subscale of interest at each time point on the variable of interest. For the first randomisation, this was the allocated induction treatment (intensive: CTD *versus* CVAD, non‐intensive: CTDa *versus* MP) adjusting for the baseline value of the subscale, the bisphosphonate allocation (sodium clodronate or zoledronic acid), ISS (I‐III), age, sex, haemoglobin at baseline (<115, ≥115 g/l for males; <95, ≥95 g/l for females), corrected serum calcium at baseline (<2·6, ≥2·6 mmol/l), serum creatinine at baseline (<140, ≥140 μmol/l) and platelet count at baseline (<150, ≥150 × 10^9^/l). Similarly, for the maintenance randomisation, the variable of interest was the randomised treatment allocation (thalidomide *versus* observation only) adjusting for the baseline value of the subscale, the treatment group allocated by the first randomisation, ISS (I–III), age and sex. The assumptions of the linear regression models were investigated for each comparison. All statistical tests were two‐sided and assessed at the 5% level of significance. As a guide for interpretation, an arbitrary difference of ≥10 points in a subscale was pre‐defined as the minimal important difference (MID) required to suggest clinical relevance (Osoba *et al*, 1998).

### Sensitivity and exploratory analysis

Sensitivity analysis assessed the impact of imputation by estimating models on complete case information. Exploratory analysis consisted of graphical displays, which summarized all questionnaire subscales with respect to the induction treatments and bisphosphonate using complete case information.

## Results

### Patient progress and baseline characteristics

**Figure 1:**
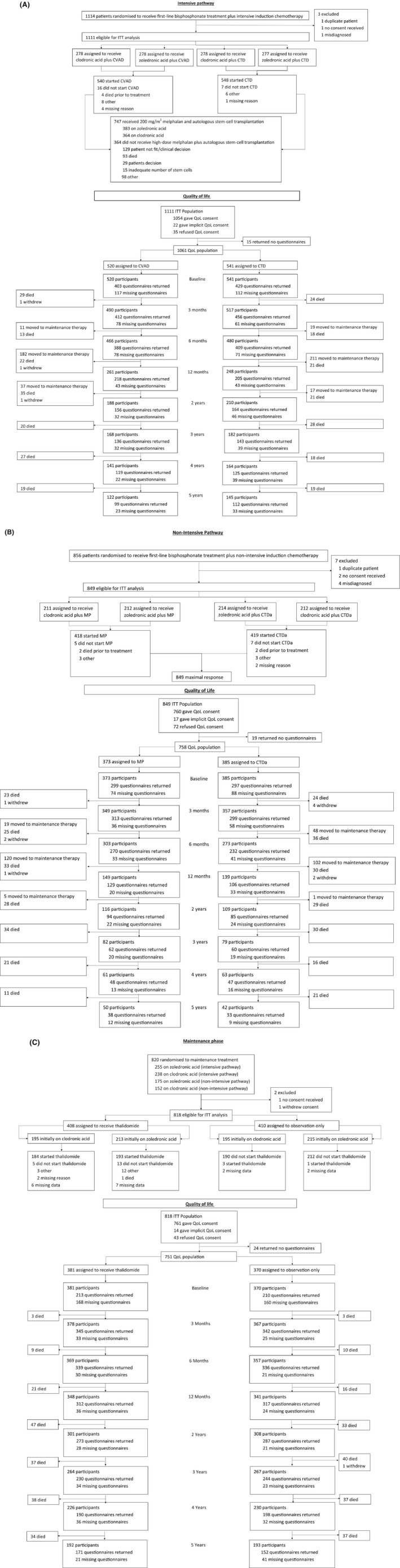
CONSORT diagrams. For the intensive pathway (A), non‐intensive pathway (B) and the maintenance phase (C). CTD, cyclophosphamide, thalidomide and dexamethasone; CTDa, attenuated cyclophosphamide, thalidomide and dexamethasone; CVAD, cyclophosphamide, vincristine, doxorubicin and dexamethasone; ITT, intention to treat; MP, melphalan and prednisolone; QoL, quality of life.

Overall 1970 patients were recruited to Myeloma IX; 1822 (92·49%) of these were included in the QoL sub‐study. The progress of patients through the sub‐study is shown for the 1819 participants at first randomisation (*N* = 1061 intensive pathway, *N* = 758 non‐intensive pathway) and for the 751 patients at maintenance randomisation in the CONSORT diagrams (Fig 1). In each population intensive and non‐intensive pathways [Table 1 (key demographics and subscales of special interest), Table SI (haematological characteristics and remaining subscales)] and maintenance phase (Table SII) the distributions of age, gender, ISS and baseline QoL were similar by allocated treatment. The average ages of patients in the intensive pathway, non‐intensive pathway and maintenance phase were 58, 73 and 63 years respectively, the latter figure reflecting the inclusion of patients from the two pathways.

**Table 1:** Select baseline demographics of the QoL population for patients in the intensive and non‐intensive pathway. The full haematological characteristics and remaining subscales can be found within Table SI

	Intensive pathway			Non‐intensive pathway		
	CVAD (*n* = 520)	CTD (*n* = 541)	Total (*n* = 1061)	MP (*n* = 373)	CTDa (*n* = 385)	Total (*n* = 758)
Age (years) at first randomisation, mean (SD)	57·7 (7·39)	57·9 (7·47)	57·8 (7·43)	73·3 (5·74)	73·1 (5·00)	73·2 (5·37)
Sex, *n* (%)						
Male	324 (62·3%)	336 (62·1%)	660 (62·2%)	204 (54·7%)	217 (56·4%)	421 (55·5%)
Female	196 (37·7%)	205 (37·9%)	401 (37·8%)	169 (45·3%)	168 (43·6%)	337 (44·5%)
Race, *n* (%)						
Caucasian	510 (98·1%)	514 (95·0%)	1024 (96·5%)	365 (97·9%)	373 (96·9%)	738 (97·4%)
Black African	2 (0·4%)	7 (1·3%)	9 (0·8%)	1 (0·3%)	2 (0·5%)	3 (0·4%)
Black Caribbean	2 (0·4%)	3 (0·6%)	5 (0·5%)	4 (1·1%)	3 (0·8%)	7 (0·9%)
Asian	4 (0·8%)	9 (1·7%)	13 (1·2%)	1 (0·3%)	1 (0·3%)	2 (0·3%)
Other	1 (0·2%)	7 (1·3%)	8 (0·8%)	1 (0·3%)	3 (0·8%)	4 (0·5%)
Missing data	1 (0·2%)	1 (0·2%)	2 (0·2%)	1 (0·3%)	3 (0·8%)	4 (0·5%)
ISS, *n* (%)						
I	122 (23·5%)	150 (27·7%)	272 (25·6%)	63 (16·9%)	43 (11·2%)	106 (14·0%)
II	177 (34·0%)	185 (34·2%)	362 (34·1%)	139 (37·3%)	146 (37·9%)	285 (37·6%)
III	166 (31·9%)	157 (29·0%)	323 (30·4%)	141 (37·8%)	145 (37·7%)	286 (37·7%)
Missing data	55 (10·6%)	49 (9·1%)	104 (9·8%)	30 (8·0%)	51 (13·2%)	81 (10·7%)
Pain						
Mean (SD)	53·3 (34·41)	53·6 (35·60)	53·4 (35·01)	52·3 (34·46)	50·0 (35·76)	51·2 (35·10)
Missing	118	112	230	74	89	163
Fatigue						
Mean (SD)	50·2 (29·36)	50·2 (29·36)	50·2 (29·36)	50·1 (27·91)	50·0 (29·88)	50·1 (28·89)
Missing	118	112	230	78	88	166
Physical Functioning						
Mean (SD)	61·5 (30·07)	59·6 (31·04)	60·5 (30·57)	56·5 (27·72)	58·1 (29·34)	57·3 (28·53)
Missing	117	112	229	76	89	165
Global Health Status /Quality of Life						
Mean (SD)	51·2 (27·58)	48·6 (27·48)	49·9 (27·54)	49·7 (24·49)	51·6 (27·10)	50·6 (25·82)
Missing	118	117	235	79	94	173

CTD, cyclophosphamide, thalidomide and dexamethasone; CTDa, attenuated cyclophosphamide, thalidomide and dexamethasone; CVAD, cyclophosphamide, vincristine, doxorubicin and dexamethasone; MP, melphalan and prednisolone; SD, standard deviation; ISS, international staging system.

### Questionnaire return

The CONSORT diagrams show the questionnaire return rate by allocated treatment group (Fig 1). The return rate and completeness of the questionnaires were generally good with a lower return rate at baseline (20% missing baseline questionnaires in both the intensive and non‐intensive pathways and 44% missing in the maintenance phase). In the intensive pathway, the CVAD group had a fairly constant rate of ‘missingness’, which contrasted with the CTD group where a large discrepancy was observed at 4 years (CVAD: 16% missing, CTD: 24% missing). In the non‐intensive pathway, a similar proportion of questionnaires were returned in both groups at each time point with the exception of 12 months post‐randomisation (MP: 13% missing, CTDa: 24% missing). In the maintenance phase, the return rate improved after the low return rate at baseline, with only approximately 10% of questionnaires missing up to 3 years post‐randomisation. The highest percentage of total ‘missingness’ not relating to a conditional question was 63%, 71% and 48% for the intensive and non‐intensive pathways and maintenance phase, respectively. Thus 71 imputed datasets were created for each analysis population.

### Principal findings in the treatment pathways

**Figure 2:**
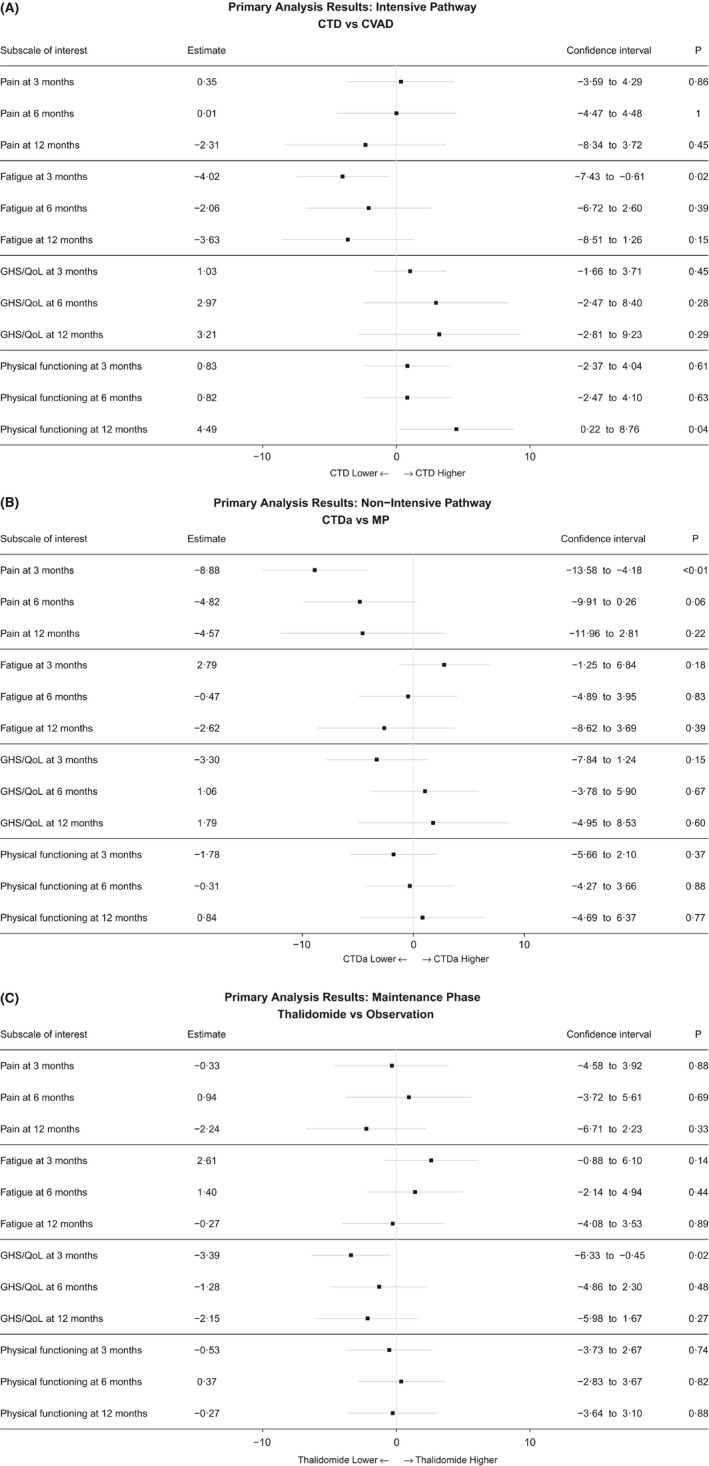
Forest plots of the primary analysis results. For the intensive pathway (A) and non‐intensive pathway (B) the models were adjusted for the baseline value of the subscale, the bisphosphonate allocation, International Staging System (ISS), age, sex and the stratification factors of the first randomisation. For the maintenance phase (C), the models were adjusted for the baseline value of the subscale, the treatment group allocated by the first randomisation, ISS, age and sex. Note that Pain and Fatigue are worse with higher scores whereas Global Health Status/Quality of Life and Physical Functioning are better with higher scores. The black squares and horizontal lines represent the treatment estimate and the associated 95% confidence interval, respectively. CTD, cyclophosphamide, thalidomide and dexamethasone; CTDa, attenuated cyclophosphamide, thalidomide and dexamethasone; CVAD, cyclophosphamide, vincristine, doxorubicin and dexamethasone; GHS/QoL, Global Health Status/Quality of Life; MP, melphalan and prednisolone.

In the intensive pathway there were no significant differences at any time point between treatment with CVAD and CTD with respect to Pain and Global Health Status/Quality of Life (Fig 2A). However, there were small significant differences in favour of CTD for Fatigue at 3 months with a persistent trend for benefit at 6 and 12 months (Fatigue lower with CTD, 3 months: −4·02 [95% confidence interval (CI) −7·43, −0·61], *P* = 0·02; 6 months: −2·06 [95% CI −6·72, 4·48], *P* = 0·39; 12 months: −3·63 [95% CI −8·51, 1·26], *P* = 0·15, Fig 2A) and Physical Functioning at 12 months after small non‐significant benefits at 3 and 6 months (Physical Functioning higher with CTD, 3 months: 0·83 [95% CI −2·37, 4·04], *P* = 0·61; 6 months: 0·82 [95% CI −2·47, 4·10], *P* = 0·63; 12 months: 4·49 [95% CI 0·22, 8·76], *P* = 0·04, Fig 2A).

In the non‐intensive pathway there was a small but highly significant difference in favour of CTDa for Pain at 3 months with a persistent trend for benefit at 6 and 12 months (Pain lower for CTDa, 3 months: −8·88 [95% CI −13·58, −4·18], *P* = 0·0002; 6 months: −4·82 [95% CI −9·91, 0·26], *P* = 0·06; 12 months: −4·57 [95% CI −11·96, 2·81], *P* = 0·22, Fig 2B). This difference was smaller than our pre‐specified MID. There were no significant differences for Fatigue, Global Health Status/Quality of Life or Physical Functioning (Fig 2B).

In the maintenance phase there was a small significant difference in favour of observation only for Global Health Status/Quality of Life at 3 months with a persistent trend for detriment at 6 months and 12 months (Global Health Status/Quality of Life lower with thalidomide maintenance, 3 months: −3·39 [95% CI −6·33, −0·45], *P* = 0·02; 6 months: −1·28 [95% CI −4·86, 2·30], *P* = 0·48; 12 months: −2·15 [95% CI −5·98, 1·67], *P* = 0·27, Fig 2C). There were no significant differences for Pain, Fatigue or Physical Functioning (Fig 2C).

There was no indication of strong violations of the modelling assumptions. Any observed violations were consistent within each imputed dataset as well as in the complete case analysis.

### Long‐term QoL in subscales of special interest

**Figure 3:**
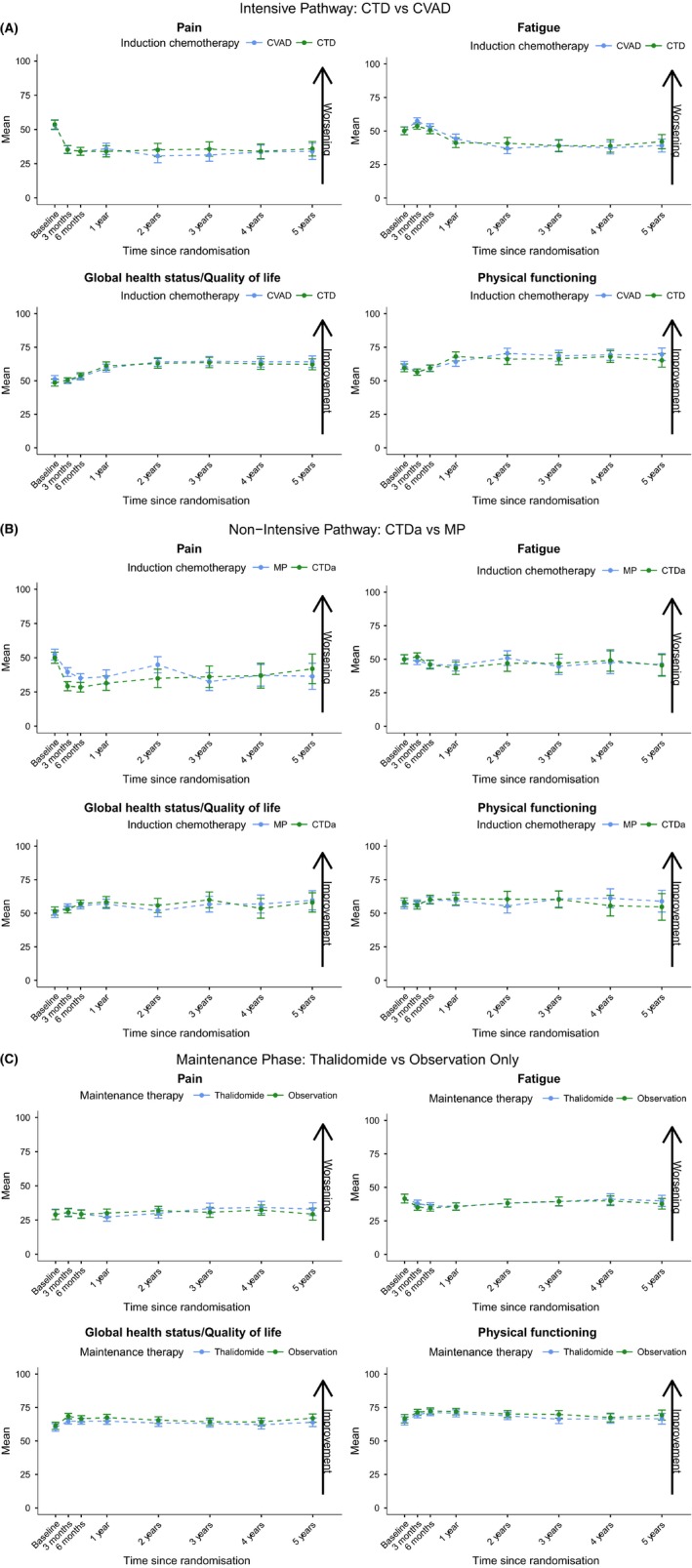
Descriptive plots for the exploratory analysis showing the subscales; Pain, Fatigue, Global Health Status/Quality of Life and Physical Functioning. For the intensive pathway (A), non‐intensive pathway (B) and maintenance phase (C). These graphs present the mean subscale scores and approximate 95% confidence intervals at each time point by the variable of interest for the four key subscales in complete case data. Note that Pain and Fatigue are worse with higher scores whereas Global Health Status/Quality of Life and Physical Functioning are better with higher scores. CTD, cyclophosphamide, thalidomide and dexamethasone; CTDa, attenuated cyclophosphamide, thalidomide and dexamethasone; CVAD, cyclophosphamide, vincristine, doxorubicin and dexamethasone; MP, melphalan and prednisolone.

In the longer‐term, at 2, 3, 4 and 5 years post‐randomisation, the pre‐specified subscales of Pain, Fatigue, Global Health Status/Quality of Life and Physical Functioning show few differences between the allocated treatments in the intensive pathway, non‐intensive pathway or maintenance phase. In the intensive pathway (Fig 3A) there is a relatively short‐term negative effect on both Fatigue and Physical Functioning that could be attributable to high‐dose therapy and ASCT. However, these negative effects do not persist beyond 1 year of follow‐up and do not translate to Global Health Status/Quality of Life, which has a general increasing trend, or to any of the other subscales. There are no clear temporal trends in the subscales of special interest for either the non‐intensive pathway or maintenance phase (Fig 3B and C respectively).

### Short and long‐term QoL in other subscales

Other EORTC QLQ‐C30 and QLQ‐MY24 subscales show largely similar trends and trajectories by allocated treatment. In the intensive pathway CVAD patients had worse Diarrhoea, Insomnia and Side Effects compared to CTD patients at 3 months. It appeared that patients in the CTD group had worse Constipation than patients on CVAD at 3 months (Figure S1A). In the non‐intensive pathway, patients on CTDa had worse Constipation than patients on MP at 3 months (Figure S1B). In the maintenance phase, Constipation was worse in those on thalidomide maintenance therapy compared to observation only both at 3 and 6 months (Figure S1C).

### Short and long‐term QoL with respect to allocated bisphosphonate treatment

There was no clear evidence of a difference in any subscale with respect to allocated bisphosphonate treatment, as all of the confidence intervals overlapped at each time point within each subscale (Figure S2).

## Discussion

The Myeloma IX study evaluated the use of an immunomodulatory agent (as both induction and maintenance treatment), zoledronic acid (a newer bisphosphonate agent that required parenteral treatment) and high dose therapy with ASCT (without entry criteria explicitly connected to patient age) and showed a number of positive clinical outcomes with improved overall response, progression‐free survival (PFS) and overall survival (OS). The analysis in this report has shown that these improvements have not come at the cost of clinically relevant decreases in patient‐reported HR‐QoL over the short‐term (during initial treatment) or in the long‐term (during maintenance treatment and subsequent follow‐up).

For the primary analysis, the only factor to demonstrate a clear and highly statistically significant difference between treatments was the Pain result at 3 months, in favour of CTDa (*P* = 0·0002), this result being supported by a persistent trend in benefit at 6 and 12 months. Although there were statistically significant differences at the 5% level in Fatigue at 3 months and Physical Functioning at 12 months, in favour of CTD, and in Global Health Status/Quality of Life at 3 months in the maintenance phase in favour of observation only compared with thalidomide maintenance, these differences were all slight, and should be interpreted with caution given the number of tests performed.

It is interesting that pain was reduced with CTDa compared with MP, especially in the short term. It is apparent that the treatment that is more active against the myeloma is also the most effective at relieving pain, despite potentially having greater toxicity. Although the therapies used in Myeloma IX have been largely superseded by more active agents, this finding is still highly relevant to current studies, and is important to explain to the patient, given that, although they are receiving potentially more toxic drugs, the greater activity of these drugs will render them more effective, both in terms of treating the myeloma itself, but also potentially in terms of relieving pain. The same applies to the other HR‐QoL measures, namely fatigue and physical functioning, which appeared to be improved with CTD/CTDa.

In terms of clinical relevance, according to the pre‐defined MID, the primary analysis showed no ‘clinically relevant’ differences. However, adopting alternative guidance on the interpretation of the individual elements of the EORTC QLQ‐C30 questionnaire suggests that all but Pain at 3 months, which favoured CTDa in the non‐intensive pathway, was unlikely to have much clinical relevance (Cocks *et al*, 2011). This same interpretation would have been the conclusion had we adopted alternative criteria where anything between a 6‐ and 17‐point difference in any sub‐scale is regarded as clinically relevant (Kvam *et al*, 2010).

However, if these results are considered temporally and in the long‐term, most of the subscales are in favour of the experimental treatment. For example, Global Health Status/Quality of Life in the intensive pathway increases in favour of CTD as time passes, despite the difference not attaining statistical or clinical significance according to our pre‐determined MID. This raises the question, what is more important: a large change in an individual scale, or multiple small changes across key subscales that are maintained over time? Alternative definitions of a MID, whether it be ≥5 points (Kvam *et al*, 2010; Verelst *et al*, 2011; Delforge *et al*, 2012), subscale specific (Cocks *et al*, 2011; Dimopoulos *et al*, 2014; Delforge *et al*, 2015), or ≥10 points, as used in this work, makes comparison between studies very difficult and research less meaningful. A consensus on such measurements is clearly required.

In our study, absence of significant differences at each time point during follow up indicates that there is little perturbation of HR‐QoL in the intensive pathway, when comparing the influence of CTD and CVAD, in the non‐intensive pathway, when investigating the difference between CTDa and MP, or in the maintenance phase when examining the impact of thalidomide maintenance therapy compared to observation only. Also, so far as the two bisphosphonates are concerned, the informal comparisons suggest little difference in HR‐QoL between patients taking clodronate and those receiving zoledronic acid.

These results are reassuring and supportive of our conclusions concerning the improved responses and survival outcomes following treatment in Myeloma IX. In the intensive pathway, CTD improved the overall response rate compared to CVAD and CTD was shown to be non‐inferior to CVAD in terms of PFS and OS (Morgan *et al*, 2012a). In the non‐intensive pathway, CTDa improved response rate over MP and CTDa was shown to significantly improve PFS compared to MP (Morgan *et al*, 2011). In the maintenance phase, thalidomide was shown to improve PFS and was associated with improved OS compared to observation only although the median duration of maintenance therapy was only 9 months (Morgan *et al*, 2012c). Zoledronic acid reduced the risk of skeletal‐related events (Morgan *et al*, 2012b) and significantly improved OS compared to sodium clodronate (Morgan *et al*, 2010). These gains in response and outcome were accompanied by some improvements rather than impairment of QoL, as determined by current HR‐QoL measurement. However, the trial setting and the relatively short periods of maintenance thalidomide delivered, median treatment duration was 7 months (range, 0–50 months) overall and 9 (range, 0–50 months) and 6 (range, 0–46 months) months in the intensive and non‐intensive pathways, respectively (Morgan *et al*, 2012c), preclude any firm conclusions about the longer term impact of cumulative neuropathic and other side effects on HR‐QoL.

These findings are broadly similar to the few published reports of comparable approaches to treatment. The VISTA trial (Delforge *et al*, 2012) reported that QoL was comparable between the two arms (bortezomib and MP (VMP) *versus* MP) after approximately 1 year in a population similar to the non‐intensive pathway. The finding that thalidomide maintenance therapy has little impact on QoL is consistent with an earlier study (Verelst *et al*, 2011) and the combination of lenalidomide and low‐dose dexamethasone (RD) *versus* MPT has been reported to improve OS without having a negative effect to QoL (Delforge *et al*, 2015).

A limitation on all HR‐QoL studies has been the high proportion of missing data. Although the returned questionnaires were largely complete, there was a substantial number of questionnaires missing at each time point, the baseline questionnaire having the worst return rate. To avoid the exclusion of a large proportion of patient data at later time points, MICE was implemented to impute any missing question answers or baseline measurements. MICE was chosen as it is a suitable stochastic method which is easy to implement (https://stats.idre.ucla.edu/sas/seminars/multiple-imputation-in-sas/mi_new_1/), and has been applied in a number of recent clinical trials (Ali *et al*, 2013; Coates *et al*, 2015; Neoptolemos *et al*, 2017). MICE assumes that the missing data are missing at random (MAR). i.e., the data is missing conditional on the information that is observed. MAR is an untestable assumption; however, it was thought to be acceptable as the questionnaires were administered at definitive (randomisation‐based) rather than subjective (treatment‐ or disease‐based) time points. In addition, sensitivity analyses also showed that results obtained using MICE were consistent with those obtained in a complete case analysis. An alternative criticism could be the choice to exclude any corrections for multiplicity. As only a subset of the total number of endpoints (4 out of 20) and time points (3 out of 7) were pre‐specified and modelled as of primary interest (generating 36 out of a possible 1020 tests) it was deemed not necessary. However, had we adjusted for the 36 simultaneous tests the results and conclusions would not have deviated from those discussed here given the small number of statistically significant results.

Patient‐reported HR‐QoL has been considered an important endpoint for patients with MM (Kvam & Waage, 2015), giving the patient input on the effects of treatment (Delforge *et al*, 2012). The results of this study showed that improvements in clinical outcomes were not at the detriment of patient reported HR‐QoL. The findings are reassuring in the context of continuing development of sequential treatment for induction, consolidation and maintenance. However, such large‐scale studies, this being the largest to date, are a major undertaking and very unlikely to be given priority in future studies. Indeed, it could be that more sensitive QoL instruments, or potentially instruments focussed on specific domains, for example neurological, will be required to identify clinically relevant differences between treatment combinations. In the context of current trends in treatment with combinations of immunomodulatory agents, proteasome inhibitors, monoclonal antibodies and HDAC inhibitors, be it in modules of intensification as consolidation intended to achieve MRD negativity, sequential, multi‐agent treatment as longer term “maintenance”, or both, there will be a need to monitor the impact on patients’ QoL. New standardised, ideally simplified, approaches that consider specific side effects of given agents as well as key QoL measures should be explored.

## Authorship contribution

JAC, GJM and GHJ were the chief investigators, SEB, JAC, GJM, GHJ, FED and MTD designed the trial and developed the protocol. K‐LR, WMG, DAC and JAC conducted the study, analysed the data and drafted the manuscript. All authors reviewed and approved the final manuscript.

## Disclaimers

The views or opinions expressed in this manuscript are not a representation of the views or opinions of the study funders, sponsor or the authors’ institutions.

## Conflict of interest

K‐LR: Research Funding – Amgen, Celgene, Merck. WMG: Research Funding – Amgen, Celgene, Merck. Honoraria – Janssen Corp; Consulting or Advisory Role – Celgene. DAC: Research Funding – Amgen, Celgene, Merck. SB: Research Funding – Amgen, Celgene, Merck. GC: Consulting or Advisory Role – Takeda, Celgene, Janssen Corp; Research Funding: Takeda, Janssen Corp. RGO: Honoraria – Takeda, Celgene, Janssen Corp; Consulting or Advisory Role – Celgene, Janssen Corp; Research Funding – Celgene; Travel, Accommodations, Expenses – Takeda, Janssen Corp. MTD: Stock and Other Ownership Interests – Abingdon Health; Consulting or Advisory Role – Abingdon Health. FED: Honoraria – Takeda, Janssen Corp, Celgene, Janssen Corp; Consulting or Advisory Role – Takeda, Celgene, Janssen Corp; Research Funding: Amgen, Celgene; Travel, Accommodations, Expenses: Takeda, Celgene. GJM: Honoraria – Takeda, Celgene; Consulting or Advisory Role – Celgene, Takeda, Bristol Meyers; Research Funding – Celgene, Janssen Corp. GJH: Honoraria – Takeda, Amgen, Celgene, Janssen Corp; Consulting or Advisory Role – Takeda, Amgen, Celgene, Janssen Corp; Research Funding: Amgen, Celgene; Travel, Accommodations, Expenses: Takeda, Celgene. JAC: Research Funding; Celgene.

## Supporting information


**Table SI.** Additional baseline demographics of the QoL population for the patients in the intensive and non‐intensive pathways.
**Table SII.** Baseline demographics of the QoL population for patients in the maintenance phase.
**Fig S1.** Remaining descriptive plots for the three analysis populations. Intensive (A) non‐intensive (B) and maintenance (C). These graphs present the mean subscale scores and approximate 95% confidence intervals at each time point by the variable of interest for complete case data.
