# Supplementary material for: Quality of life during and following sequential treatment of previously untreated patients with multiple myeloma: findings of the Medical Research Council Myeloma IX randomised study
Source: Br J Haematol. 2018 Jul 9;182(6):816–29. doi: 10.1111/bjh.15459 (PMC6175065; doi:10.1111/bjh.15459)
Supplement: Supplementary file 1 — Table SI. Additional baseline demographics of the QoL population for the patients in the intensive and non‐intensive pathways. Table SII. Baseline demographics of the QoL population for patients in the maintenance phase. Fig S1. Remaining descriptive plots for the three analysis populations. Intensive (A) non‐intensive (B) and maintenance (C). These graphs present the mean subscale scores and approximate 95% confidence intervals at each time point by the variable of interest for complete case data. [file BJH-182-816-s001.docx]

***Table SI: Additional baseline demographics of the QoL population for the patients in the intensive and non-intensive pathways.***

|  | **Intensive Pathway** | | | **Non-Intensive Pathway** | | |
| --- | --- | --- | --- | --- | --- | --- |
|  | **CVAD (n=520)** | **CTD (n=541)** | **Total (n=1061)** | **MP (n=373)** | **CTDa (n=385)** | **Total (n=758)** |
| **Haemoglobin at Baseline, n (%)** |  |  |  |  |  |  |
| Low (less than 9.5/11.5g/dl females/males) | 238 (45.8%) | 254 (47.0%) | 492 (46.4%) | 181 (48.5%) | 189 (49.1%) | 370 (48.8%) |
| High  (greater than or equal to 9.5/11.5g/dl females/males) | 282 (54.2%) | 287 (53.0%) | 569 (53.6%) | 191 (51.2%) | 195 (50.6%) | 386 (50.9%) |
| Missing Data | 0 (0.0%) | 0 (0.0%) | 0 (0.0%) | 1 (0.3%) | 1 (0.3%) | 2 (0.3%) |
| **Serum Creatinine at Baseline, n (%)** |  |  |  |  |  |  |
| Low (below 140μmol/l) | 412 (79.2%) | 423 (78.2%) | 835 (78.7%) | 290 (77.7%) | 300 (77.9%) | 590 (77.8%) |
| High (greater than or equal to 140μmol/l) | 108 (20.8%) | 118 (21.8%) | 226 (21.3%) | 82 (22.0%) | 84 (21.8%) | 166 (21.9%) |
| Missing Data | 0 (0.0%) | 0 (0.0%) | 0 (0.0%) | 1 (0.3%) | 1 (0.3%) | 2 (0.3%) |
| **Corrected Serum Calcium at Baseline, n (%)** |  |  |  |  |  |  |
| Low (less than 2.6 mmol/l) | 384 (73.8%) | 396 (73.2%) | 780 (73.5%) | 282 (75.6%) | 296 (76.9%) | 578 (76.3%) |
| High (greater than or equal to 2.6mmol/l) | 136 (26.2%) | 145 (26.8%) | 281 (26.5%) | 90 (24.1%) | 88 (22.9%) | 178 (23.5%) |
| Missing Data | 0 (0.0%) | 0 (0.0%) | 0 (0.0%) | 1 (0.3%) | 1 (0.3%) | 2 (0.3%) |
| **Platelets at Baseline, n (%)** |  |  |  |  |  |  |
| Low (below 150x10^9/l) | 66 (12.7%) | 71 (13.1%) | 137 (12.9%) | 49 (13.1%) | 54 (14.0%) | 103 (13.6%) |
| High (greater than or equal to 150x10^9/l) | 454 (87.3%) | 470 (86.9%) | 924 (87.1%) | 323 (86.6%) | 330 (85.7%) | 653 (86.1%) |
| Missing Data | 0 (0.0%) | 0 (0.0%) | 0 (0.0%) | 1 (0.3%) | 1 (0.3%) | 2 (0.3%) |
| **Paraprotein type, n (%)** |  |  |  |  |  |  |
| IgG | 311 (59.8%) | 325 (60.1%) | 636 (59.9%) | 228 (61.1%) | 223 (57.9%) | 451 (59.5%) |
| IgA | 113 (21.7%) | 115 (21.3%) | 228 (21.5%) | 89 (23.9%) | 91 (23.6%) | 180 (23.7%) |
| IgM | 3 (0.6%) | 2 (0.4%) | 5 (0.5%) | 1 (0.3%) | 2 (0.5%) | 3 (0.4%) |
| No paraprotein | 10 (1.9%) | 9 (1.7%) | 19 (1.8%) | 4 (1.1%) | 6 (1.6%) | 10 (1.3%) |
| IgD | 11 (2.1%) | 12 (2.2%) | 23 (2.2%) | 3 (0.8%) | 10 (2.6%) | 13 (1.7%) |
| Light chain only | 68 (13.1%) | 71 (13.1%) | 139 (13.1%) | 43 (11.5%) | 49 (12.7%) | 92 (12.1%) |
| Missing Data | 4 (0.8%) | 7 (1.3%) | 11 (1.0%) | 5 (1.3%) | 4 (1.0%) | 9 (1.2%) |
| **Light Chain Type, n (%)** |  |  |  |  |  |  |
| Lambda | 163 (31.3%) | 166 (30.7%) | 329 (31.0%) | 124 (33.2%) | 124 (32.2%) | 248 (32.7%) |
| Kappa | 307 (59.0%) | 334 (61.7%) | 641 (60.4%) | 224 (60.1%) | 231 (60.0%) | 455 (60.0%) |
| Missing Data | 50 (9.6%) | 41 (7.6%) | 91 (8.6%) | 25 (6.7%) | 30 (7.8%) | 55 (7.3%) |
| **Social Functioning** |  |  |  |  |  |  |
| Mean (SD) | 53.4 (36.58) | 49.4 (37.41) | 51.3 (37.04) | 57.5 (36.49) | 57.4 (37.10) | 57.5 (36.76) |
| Missing | 119 | 117 | 236 | 78 | 90 | 168 |
| **Cognitive Functioning** |  |  |  |  |  |  |
| Mean (SD) | 73.1 (27.01) | 74.8 (26.82) | 74.0 (26.91) | 74.7 (24.08) | 74.8 (26.96) | 74.8 (25.54) |
| Missing | 118 | 115 | 233 | 77 | 89 | 166 |
| **Emotional Functioning** |  |  |  |  |  |  |
| Mean (SD) | 65.0 (25.28) | 65.8 (24.97) | 65.4 (25.11) | 73.6 (22.96) | 73.3 (23.02) | 73.4 (22.97) |
| Missing | 118 | 115 | 233 | 77 | 89 | 166 |
| **Constipation** |  |  |  |  |  |  |
| Mean (SD) | 32.2 (35.74) | 30.6 (35.91) | 31.4 (35.81) | 37.1 (36.81) | 36.3 (37.42) | 36.7 (37.09) |
| Missing | 121 | 116 | 237 | 79 | 89 | 168 |
| **Nausea / Vomiting** |  |  |  |  |  |  |
| Mean (SD) | 14.1 (21.24) | 12.3 (22.10) | 13.2 (21.70) | 13.5 (20.91) | 14.4 (22.29) | 13.9 (21.59) |
| Missing | 119 | 112 | 231 | 75 | 88 | 163 |
| **Insomnia** |  |  |  |  |  |  |
| Mean (SD) | 39.8 (32.95) | 40.8 (34.99) | 40.3 (34.00) | 35.0 (34.45) | 33.1 (33.22) | 34.1 (33.83) |
| Missing | 119 | 113 | 232 | 74 | 92 | 166 |
| **Financial Problems** |  |  |  |  |  |  |
| Mean (SD) | 20.8 (32.38) | 25.2 (35.43) | 23.1 (34.04) | 8.0 (18.01) | 12.1 (26.70) | 10.1 (22.86) |
| Missing | 121 | 117 | 238 | 80 | 91 | 171 |
| **Dyspnoea** |  |  |  |  |  |  |
| Mean (SD) | 28.4 (31.11) | 28.6 (29.97) | 28.5 (30.51) | 33.7 (32.18) | 32.7 (30.84) | 33.2 (31.50) |
| Missing | 119 | 115 | 234 | 77 | 92 | 169 |
| **Appetite loss** |  |  |  |  |  |  |
| Mean (SD) | 31.3 (33.15) | 30.9 (33.90) | 31.1 (33.52) | 31.9 (34.95) | 35.4 (35.46) | 33.6 (35.22) |
| Missing | 118 | 112 | 230 | 75 | 88 | 163 |
| **Diarrhoea** |  |  |  |  |  |  |
| Mean (SD) | 9.4 (20.49) | 8.0 (20.06) | 8.7 (20.27) | 9.1 (21.35) | 8.7 (19.33) | 8.9 (20.35) |
| Missing | 119 | 116 | 235 | 79 | 90 | 169 |
| **Disease Symptoms** |  |  |  |  |  |  |
| Mean (SD) | 38.3 (24.43) | 39.7 (24.47) | 39.1 (24.44) | 37.2 (23.11) | 37.3 (26.22) | 37.3 (24.70) |
| Missing | 119 | 116 | 235 | 80 | 89 | 169 |
| **Social Support** |  |  |  |  |  |  |
| Mean (SD) | 94.2 (14.69) | 94.0 (14.86) | 94.1 (14.77) | 91.9 (19.31) | 93.5 (17.95) | 92.7 (18.64) |
| Missing | 128 | 124 | 252 | 83 | 93 | 176 |
| **Body Image** |  |  |  |  |  |  |
| Mean (SD) | 24.1 (33.71) | 23.2 (32.05) | 23.7 (32.85) | 22.9 (34.58) | 20.3 (32.21) | 21.6 (33.41) |
| Missing | 125 | 122 | 247 | 86 | 99 | 185 |
| **Future Perspective** |  |  |  |  |  |  |
| Mean (SD) | 63.2 (26.23) | 61.0 (25.35) | 62.0 (25.79) | 48.5 (27.22) | 47.9 (25.89) | 48.2 (26.54) |
| Missing | 119 | 117 | 236 | 79 | 93 | 172 |
| **Side Effects** |  |  |  |  |  |  |
| Mean (SD) | 21.1 (16.42) | 22.0 (17.46) | 21.6 (16.96) | 21.9 (16.11) | 22.3 (17.25) | 22.1 (16.68) |
| Missing | 119 | 117 | 236 | 79 | 90 | 169 |

***Table SII:*** *Baseline demographics of the QoL population for patients in the maintenance phase.*

|  | **Thalidomide (n=381)** | **Observation only (n=370)** | **Total (n=751)** |
| --- | --- | --- | --- |
| Age at first randomisation, mean (SD) | 63.8 (9.96) | 62.2 (9.86) | 63.0 (9.94) |
| **Sex, n (%)** |  |  |  |
| Male | 228 (59.8%) | 228 (61.6%) | 456 (60.7%) |
| Female | 153 (40.2%) | 142 (38.4%) | 295 (39.3%) |
| **Race, n (%)** |  |  |  |
| Caucasian | 370 (97.1%) | 358 (96.8%) | 728 (96.9%) |
| Black African | 1 (0.3%) | 1 (0.3%) | 2 (0.3%) |
| Black Caribbean | 2 (0.5%) | 4 (1.1%) | 6 (0.8%) |
| Asian | 2 (0.5%) | 6 (1.6%) | 8 (1.1%) |
| Other | 5 (1.3%) | 0 (0.0%) | 5 (1.3%) |
| Missing Data | 1 (0.3%) | 1 (0.3%) | 2 (0.3%) |
| **International Staging System (ISS), n (%)** |  |  |  |
| I | 84 (22.0%) | 88 (23.8%) | 172 (22.9%) |
| II | 145 (38.1%) | 124 (33.5%) | 269 (35.8%) |
| III | 114 (29.9%) | 117 (31.6%) | 231 (30.8%) |
| Missing Data | 38 (10.0%) | 41 (11.1%) | 79 (10.5%) |
| **Haemoglobin at Baseline, n (%)** |  |  |  |
| Low (less than 9.5/11.5g/dl females/males) | 160 (42.0%) | 183 (49.5%) | 343 (45.7%) |
| High (greater than or equal to 9.5/11.5g/dl females/males) | 221 (58.0%) | 187 (50.5%) | 408 (54.3%) |
| **Serum Creatinine at Baseline, n (%)** |  |  |  |
| Low (below 140μmol/l) | 307 (80.6%) | 294 (79.5%) | 601 (80.0%) |
| High (greater than or equal to 140μmol/l) | 74 (19.4%) | 76 (20.5%) | 150 (20.0%) |
| **Corrected Serum Calcium at Baseline, n (%)** |  |  |  |
| Low (less than 2.6 mmol/l) | 278 (73.0%) | 284 (76.8%) | 562 (74.8%) |
| High (greater than or equal to 2.6mmol/l) | 103 (27.0%) | 86 (23.2%) | 189 (25.2%) |
| **Platelets at Baseline, n (%)** |  |  |  |
| Low (below 150x10^9/l) | 50 (13.1%) | 44 (11.9%) | 94 (12.5%) |
| High (greater than or equal to 150x10^9/l) | 331 (86.9%) | 326 (88.1%) | 657 (87.5%) |
| **Paraprotein type, n (%)** |  |  |  |
| IgG | 227 (59.6%) | 223 (60.3%) | 450 (59.9%) |
| IgA | 98 (25.7%) | 79 (21.4%) | 177 (23.6%) |
| IgM | 1 (0.3%) | 3 (0.8%) | 4 (0.5%) |
| No paraprotein | 4 (1.0%) | 9 (2.4%) | 13 (1.7%) |
| IgD | 12 (3.1%) | 5 (1.4%) | 17 (2.3%) |
| Light chain only | 37 (9.7%) | 47 (12.7%) | 84 (11.2%) |
| Missing Data | 2 (0.5%) | 4 (1.1%) | 6 (0.8%) |
| **Light Chain Type, n (%)** |  |  |  |
| Lambda | 123 (32.3%) | 117 (31.6%) | 240 (32.0%) |
| Kappa | 231 (60.6%) | 227 (61.4%) | 458 (61.0%) |
| Missing Data | 27 (7.1%) | 26 (7.0%) | 53 (7.1%) |
| **Pain** |  |  |  |
| Mean (SD) | 29.2 (28.50) | 29.0 (26.85) | 29.1 (27.66) |
| Missing | 168 | 160 | 328 |
| **Fatigue** |  |  |  |
| Mean (SD) | 41.6 (23.87) | 41.6 (24.24) | 41.6 (24.03) |
| Missing | 168 | 161 | 329 |
| **Physical Functioning** |  |  |  |
| Mean (SD) | 65.0 (23.20) | 66.6 (23.40) | 65.8 (23.28) |
| Missing | 168 | 161 | 329 |
| **Social Functioning** |  |  |  |
| Mean (SD) | 58.4 (29.72) | 57.7 (33.06) | 58.1 (31.39) |
| Missing | 169 | 160 | 329 |
| **Cognitive Functioning** |  |  |  |
| Mean (SD) | 79.2 (21.52) | 78.5 (22.75) | 78.8 (22.11) |
| Missing | 169 | 160 | 329 |
| **Emotional Functioning** |  |  |  |
| Mean (SD) | 79.2 (21.96) | 81.9 (21.81) | 80.6 (21.90) |
| Missing | 169 | 160 | 329 |
| **Global Health Status / Quality of Life** |  |  |  |
| Mean (SD) | 60.1 (20.90) | 61.3 (20.01) | 60.7 (20.45) |
| Missing | 171 | 162 | 333 |
| **Constipation** |  |  |  |
| Mean (SD) | 11.6 (22.27) | 10.0 (22.65) | 10.8 (22.44) |
| Missing | 169 | 161 | 330 |
| **Nausea / Vomiting** |  |  |  |
| Mean (SD) | 10.9 (18.47) | 10.6 (20.55) | 10.7 (19.51) |
| Missing | 168 | 161 | 329 |
| **Insomnia** |  |  |  |
| Mean (SD) | 24.5 (29.74) | 26.0 (31.00) | 25.2 (30.34) |
| Missing | 170 | 161 | 331 |
| **Financial Problems** |  |  |  |
| Mean (SD) | 20.1 (31.42) | 21.9 (32.39) | 21.0 (31.88) |
| Missing | 170 | 160 | 330 |
| **Dyspnoea** |  |  |  |
| Mean (SD) | 25.5 (29.16) | 18.8 (24.65) | 22.2 (27.21) |
| Missing | 168 | 162 | 330 |
| **Appetite loss** |  |  |  |
| Mean (SD) | 22.7 (28.44) | 22.3 (31.09) | 22.5 (29.74) |
| Missing | 168 | 162 | 330 |
| **Diarrhoea** |  |  |  |
| Mean (SD) | 8.3 (19.44) | 11.3 (22.51) | 9.8 (21.05) |
| Missing | 169 | 161 | 330 |
| **Disease Symptoms** |  |  |  |
| Mean (SD) | 21.8 (18.71) | 21.7 (16.42) | 21.8 (17.59) |
| Missing | 170 | 160 | 330 |
| **Social Support** |  |  |  |
| Mean (SD) | 92.5 (18.26) | 93.7 (16.03) | 93.1 (17.17) |
| Missing | 172 | 160 | 332 |
| **Body Image** |  |  |  |
| Mean (SD) | 30.5 (31.73) | 32.9 (35.58) | 31.7 (33.69) |
| Missing | 172 | 161 | 333 |
| **Future Perspective** |  |  |  |
| Mean (SD) | 39.4 (26.02) | 38.4 (25.70) | 38.9 (25.84) |
| Missing | 170 | 160 | 330 |
| **Side Effects** |  |  |  |
| Mean (SD) | 20.8 (16.69) | 19.9 (15.57) | 20.3 (16.13) |
| Missing | 169 | 160 | 329 |

***Figure S1:*** *Remaining descriptive plots for the three analysis populations.* *Intensive (A) non-intensive (B) and maintenance (C). These graphs present the mean subscale scores and approximate 95% confidence intervals at each time point by the variable of interest for complete case data.*

***A***

******

***B***

******

***C***

******

***Figure S2:*** *Descriptive plots for the QoL subscales split by bisphosphate allocation – first randomisation only.* *These lines graphs present the mean subscale scores and approximate 95% confidence intervals at each time point by the variable of interest in complete case data.*
